# Bone Grafting Outcomes in Smokers Undergoing High Tibial Osteotomy: A Systematic Review

**DOI:** 10.7759/cureus.36758

**Published:** 2023-03-27

**Authors:** Pushkar Joshi, Shruti Joshi, Yogesh Joshi, Pritom M Shenoy

**Affiliations:** 1 Trauma and Orthopaedics, Betsi Cadwaladr University Health Board, Warrington, GBR; 2 Advanced Musculoskeletal Medicine, St. Helens and Knowsley NHS Foundation Trust, Prescot, GBR; 3 Trauma and Orthopaedics, Betsi Cadwaladr University Health Board, Wrexham, GBR; 4 Trauma and Orthopaedics, Wrexham Maelor Hospital, Wrexham, GBR

**Keywords:** non-smokers, ex-smokers, bone grafting, smoking, osteoarthritis (oa), 10 high tibial osteotomy

## Abstract

This systematic review summarises the findings in the literature available to show outcomes of high tibial osteotomy (HTO) with bone grafting in smokers. It also studies the trend of complications, outcome measures used and overall outcomes like union, non-union or the need to perform revision surgeries. The aim is to find out if HTO done with bone grafting improves outcomes in smokers. Articles were shortlisted using Population, Intervention, Control, and Outcomes (PICO) search design and quality assessment was completed using Jadad, STROBE (Strengthening the Reporting of Observational studies in Epidemiology), Delphi, and Critical Appraisal Skills Program (CASP) followed by data extraction by two independent authors. There was union in 97.6% of smokers who received HTO with bone grafting. A case of non-union was treated with removal of metalwork and distraction osteogenesis. Three cases of unknown demographics had arthroplasty in the time frame from HTO with bone grafting to follow up. The commonest complication post surgery was metalwork causing soft tissue irritation and lateral proximal tibial cortex fracture. Following this review we can conclude that HTO with bone grafting could be considered as an option to achieve better outcomes in smokers. Bone grafting helps healing across osteotomy sites in smokers whose healing potential is poor. Autogenous Iliac crest bone grafting is ideal due to its osteoinductive and osteoconductive properties, but has the disadvantage of donor site morbidity.

## Introduction and background

High tibial osteotomy (HTO) is a commonly performed orthopaedic surgery to treat degenerative conditions of the knee. The operation aims to modify the mechanical axis that passes through the knee joint. Normally the joint line passes medial to the midline of the knee which causes excessive forces through the medial compartment of the knee resulting in wear and tear of the joint. However, the surgery involves changing the direction of the axis to slightly lateral to the midline of the knee thereby avoiding the excessive wear and tear of the joint and leads to relief of symptoms [1].

In the United Kingdom (UK), 15.8% of the population smokes, which accounts for about 7.6 million people [2]. The total number of knee osteotomy surgeries performed in the UK since 2014 till date is 621 and 1155 are still awaiting surgery/data entry [3]. This surgical procedure is rapidly gaining popularity due to increased availability of evidence and outcomes. Smoking is a well-known factor that inhibits bone healing. Smoking leads to poor bone mineral density and inadvertently makes native bone weak and delays healing [4]. Smokers are twice as likely to develop complications like delayed union, pseudo-arthrosis and prolonged time in external fixators as compared to non-smokers [5]. However the complication of infection was predominant among non-smokers. Studies have shown the outcomes of medial open wedge tibial osteotomy (MOWTO) that 50% of non-union cases were smokers and only 23% of smokers showed bony consolidation on CT scans [6]. After extensive research, several mechanisms of the toxic effect of smoking on bone healing have been outlined as reduced oxygen tension in blood, oxygen toxic radicals and metabolites in blood, reduced blood flow due to narrowing of arterioles, direct toxic effects on osteoblasts and low concentration of circulating anti-oxidants [7]. Smoking is commonly associated with delayed union and non-union at the osteotomy site.

The above literature questions the effect of surgical procedures like osteotomies in the smoking population. Hence there arises the need to explore surgical adjuncts or additional surgical procedures to aid in bone healing in smokers. Performing bone grafting as additional surgical treatment for osteotomy non union, aids to achieve bone healing [8]. Several bone grafting options are available. Autologous iliac crest bone grafting from the patient's own body carries multiple advantages like no risk of transmitting infection, easy access and being done at the same time as primary surgery. It is considered by many as the gold standard treatment due to its osteoconductive and osteoinductive properties [9]. It also has many disadvantages like donor site morbidity, limited quantity, fracture, and haematoma formation, no possibility of re-harvesting same site and nerve damage [10]. The other available options to mitigate these disadvantages are allograft (bone bank), xenograft (bone from different species), ceramic-based synthetic substitutes (hydroxyapatite coated), platelet-rich plasma, bone morphogenic proteins (BMP 2) and mesenchymal cells [11]. Native bone quality in smokers is poor; hence relying on such bone to lay new bone on scaffolds is not worthwhile [12]. 

The increasing load of young patients with osteoarthritis (OA) is a concern as most of the young population have smoking as a lifestyle habit. The fact that smoking is an important prognostic factor that decides the outcome of the surgery drove the need to establish an alternative adjunct to treatment. Treatment of young osteoarthritic knees is a grey zone with little supporting evidence on their management. All attempts aim towards avoiding a major arthroplasty surgery due to the future risks involved with relation to revision surgeries for early failure. Hence we have aimed to find the correlation between smoking and the outcome of HTO in this systematic review.

## Review

Methods

The aim is to perform a systematic review to find out if bone grafting done in smokers who have undergone medial open wedge high tibial osteotomy for medial compartment of the knee OA aids in preventing the known complications of delayed union and non-union in this high-risk group.

The objective is to create an evidence base by performing a descriptive systematic review to support the aim. The secondary outcomes of the aim are to assess time to union, conversion to total knee replacement, complications and need for revision surgery. Also conduct appraisal and analysis of literature to determine if bone grafting would be a good option in the smoking population to aid bone healing.

An extensive search of available databases was done which included Excerpta Medica database (EMBASE), Health Management Information Consortium (HMIC), American Psychological Association (PsycINFO), Ovid Medical Literature Analysis and Retrieval System Online (MEDLINE), NHS Wales full text Journals, Ovid Journal database and COCHRANE database. All databases listed were searched using the keywords and search was carried across all titles, full text and abstracts. Limits were used for searching the databases for literature only in English from 1990 to January 2018 and restricted to the keywords which were osteoarthritis, high tibial osteotomy, smoking and bone grafting. Systematic reviews, randomized controlled trials (RCTs), cohort studies, cross-sectional studies, case series, case reports, research articles and research studies were included in limits.

Studies were included in the review if they met the following criteria as medial compartment osteoarthritis of the knee, adults over 18 years of age, human studies, patients having undergone high tibial osteotomy including smokers/ex-smokers, bone grafting utilised during surgery, any osteotomy fixation technique, studies with more than one-year follow-up, literature in English, time period from 1990 to 2018 and studies with level of evidence I-IV.

Jadad scale and Critical Appraisal Skills Programme (CASP) checklist for quality assessment of randomised clinical trials and Strengthening the Reporting of Observational studies in Epidemiology (STROBE) checklist for non-randomised trials. Quality assessment of the case series included in the study has been done utilising the Delphi checklist. Jadad scoring is a quick, easy, high inter-rater reliability and quantitative method of classifying trials into high or low score studies. A systematic review of available appraisal tools concluded that Jadad has best validity and reliability [13]. Any study with a Jadad score of <2 was excluded from the review as it indicates a poor study and high risk of bias. The CASP checklist was applied to assess quality under three broad categories: validity of results, description of results and validity/generalisability of results. Only studies that sufficed the CASP checklist were sub-categorised and appraised critically. The included studies were good quality studies. CASP is an easy, quick and systematic way of critically appraising any study and has a variety of checklists for each type of study making it even easier for new researchers to quickly assess the quality of the study [14]. Modified Delphi checklist has been developed by the Institute of Health Economics (IHE) as a reliable and valid tool for quality assessment of case series, which has been a grey area for many years due to paucity of literature [15]. STROBE 2009 was essentially designed to simplify the objective of study, methodology of study, results of study and applicability of results to the general population and to the general readers. It is again a very descriptive checklist, quick and easy to conduct.

Quality of studies conducted by Passarelli et al. and Zorzi et al. have good randomisation techniques, differentiation and detailing of intervention and control and carry high reliability [16,17]. They have precisely studied the outcomes in those patients who received a bone graft (Group A) and those who did not (Group B) [16,17]. This is a major bonus point of the two studies.

Passarelli et al.'s study, even though it has a high level of evidence (Level IB), clinically has many drawbacks [16]. This study has not clearly mentioned all the complications which highlight the possibility of selective reporting bias. Also, small sample size of smokers and non-smokers is the confounding factor for this study. Power calculation was done prior to this study and IBM SPSS software was utilised for statistical analysis. This study has potential to act as a pilot study for further research. This study was a single surgeon and single institute driven.

Zorzi et al.'s study is a single surgeon and single centre study [17]. The sample size of smokers was again small in this study. Complications have been well explained. This is a high level of evidence (Level IB) study with very low risk of bias as explained in tables earlier.

Spahn et al. clearly mentioned the failure to follow up rate but followed the intention to treat principle and mentioned that pre-operative Knee injury and Osteoarthritis Outcome Score (KOOS) did not differ much from the patients included in the study [18]. This minimises the risk of attrition bias. However the technique of patient allocation remains unclear giving rise to allocation bias.

LaPrade et al. published their case series on outcomes of HTO in young and middle-aged populations [19]. This was a single surgeon operated, single centre series. This study had no conflict of interest, but its level of evidence is low. Comparatively the drop-out rate was about 20% from the study. But the author clearly describes the reason for drop-out and they were excluded from the study. The follow-up period was also less with a minimum of two years and mean of 3.6 years.

Results

**Figure 1:**
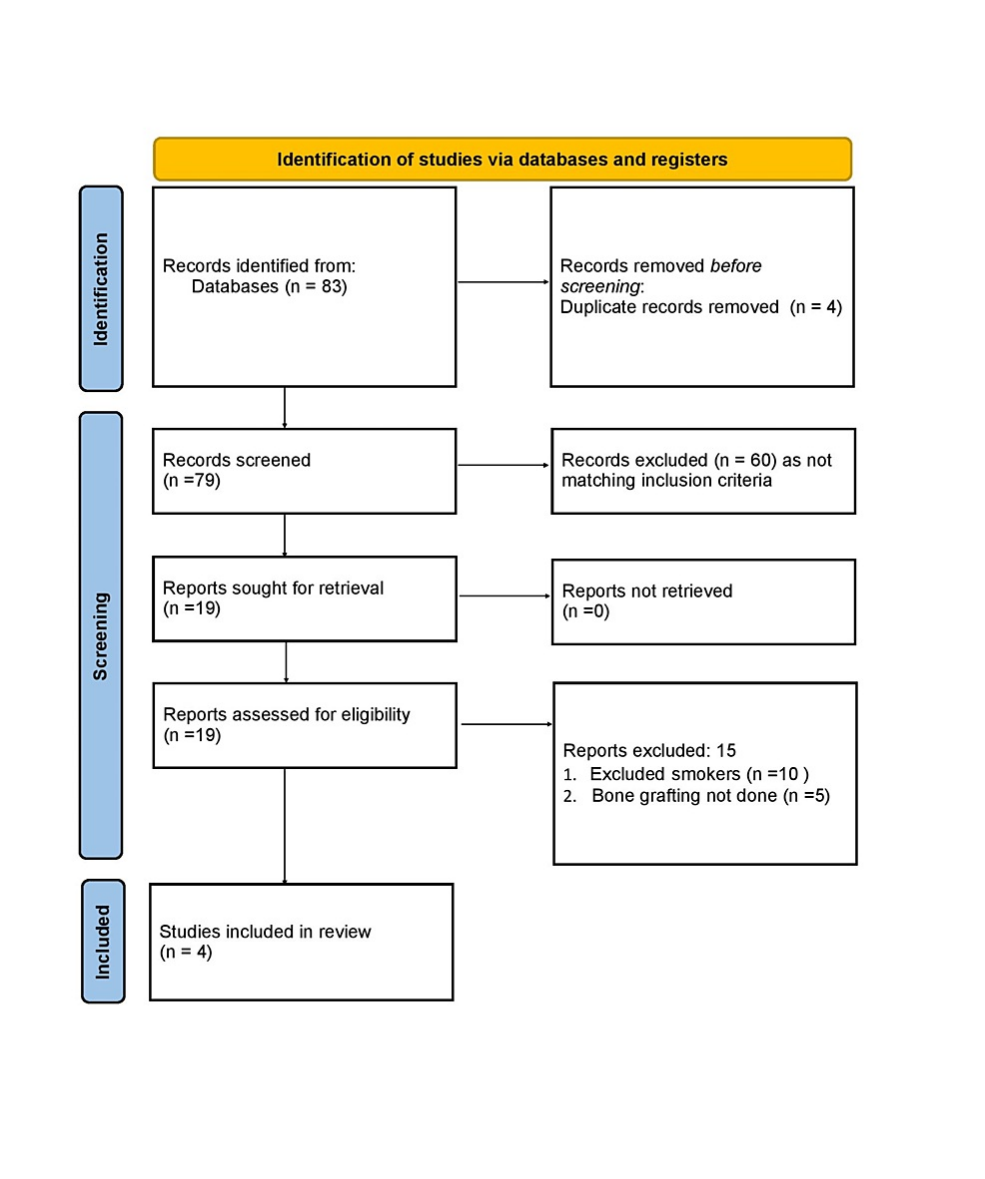
Preferred Reporting Items for Systematic Reviews and Meta-Analyses (PRISMA) flowchart for selection of studies.

The comprehensive search results are detailed below by using the Preferred Reporting Items for Systematic Reviews and Meta-Analyses (PRISMA) flow diagram (Figure 1). Data extraction was undertaken by two independent authors, thus reducing the risk of selection and reporting bias. Eighty-three records were identified after screening of databases and following removal of duplicates 79 full articles were screened. After strictly following the selection criteria, 60 studies were excluded and 19 full articles were scrutinised. However 15 further articles were excluded as the reports excluded smokers and bone grafting was not done in the treatment group. The details of included studies are displayed in Table 1. Amongst the included studies were two RCTs, one case series and one non-randomised study.

**Table 1:** Characteristics of included studies. MOWTO: Medial Open Wedge Tibial Osteotomy

Study	Date	Country	Type of study	Surgical technique	Bone graft	Type of fixation	Follow up
Passarelli et al. [16]	September 2016	Brazil	Randomized prospective clinical trial	Proximal MOWTO with plate fixation	Iliac crest bone graft	Plate	4 years
Zorzi et al. [17]	March 2010	Brazil	Randomized clinical trial	Proximal MOWTO with plate fixation	Iliac crest bone graft	Plate	1-3 years
Spahn et al. [18]	August 2005	Germany	Retrospective non-randomized study	Proximal MOWTO with plate fixation	Iliac crest bone graft	Plate	3-5 years
LaPrade et al. [19]	August 2011	North America	Prospective case series	Proximal MOWTO with plate fixation	Opteform Allograft	Plate	2-8.9 years

Outcome measures used in each of the studies were standardised. Passarelli et al. used Knee Society Score (KSS) for follow-up assessment and modified Ahlback's score for progression and staging of OA pre- and post-procedure [16]. Ahlback's scoring heavily relies on radiographic appearance and findings of the knee joint. It has poor reproducibility and reliability [20]. Similarly, KSS has poor reproducibility but the new modified KSS is highly reliable [21]. They have also measured the impact of osteotomy on the femoro-tibial angle using X-rays. Zorzi et al. assessed union at the osteotomy site by Apley and Solomon criteria for clinical and radiological union [17]. Spahn et al. developed a scoring system to assess poor prognostic factors affecting outcomes after HTO [18]. He proposed an inverse relationship between smoking and KOOS [18]. The series by LaPrade et al. used the Modified Cincinnati Knee score (MCKS) and International Knee Documentation Committee Objective Knee score (IKDCS) for outcome measurements [19]. MCKS and IKDCS have similar and high reliability, reproducibility and validity for knee cartilage conditions [22].

Complications

The complication of delayed or non-union was seen only in one patient who was a smoker in the study by LaPrade et al. [19]. All other complications could be attributed to other significant co-morbid factors like high BMI, diabetes, immune compromised state, trauma etc. Apart from Zorzi et al., none of the studies mention the time taken for union [17]. The commonest complication was fracture of the lateral cortex. The most significant one has been hardware irritation in nine patients of the LaPrade et al. series [18]. Further details are provided in Table 2.

**Table 2:** Complications post surgery DVT: Deep Venous Thrombosis

Study	Delayed union	Non union	Infection	DVT	Hardware irritation	Fracture of lateral cortex	Other
Passarelli et al. [16]	0	0	0	0	0	0	0
Zorzi et al. [17]	0	0	0	0	0	4	2
Spahn et al. [18]	0	0	2	3	0	0	2
LaPrade et al. [19]	0	1	1	0	9	2	0

The most significant endpoint for any surgical procedure is the need to revise it or convert it to a higher sophisticated surgery due to failure. Measuring the need for revising osteotomy due to non-union or need for conversion to total knee replacement (TKR) due to worsening arthritis is of utmost importance. Conversion rates to total knee replacements have been low in all the studies. It is very evident that MOWTO along with bone grafting is a good procedure with long survival rates and good outcomes even in smokers. Three patients in the study by Spahn et al. had already undergone arthroplasty between HTO and follow-up of unknown aetiology [18]. Two patients needed revision of osteotomy. These have been depicted in Table 3.

**Table 3:** Surgical conversion/revision

Study	Follow up	Conversion to arthroplasty	Osteotomy revision
Passarelli et al. [16]	4 years	0	0
Zorzi et al. [17]	1-3 years	0	0
Spahn et al. [18]	3-5 years	3	1
LaPrade et al. [19]	2-8.9 years	2	1

The results after detailed evaluation of the included studies are very promising. The most important outcome in the study by Passareli et al. was that all patients had union at the osteotomy site [16]. This indicated that all patients had a union at the osteotomy site including smokers. Zorzi et al. had a statistically significant difference in the sample size of smokers undergoing bone grafting with those undergoing no bone grafting (p<0.084) [17]. This was in spite of the small number of smokers included in the study. However, staging of OA did not correlate with outcomes (p<0.542). The healing at the osteotomy site even in smokers is assuring in spite of a small sample size. Eleven smoking patients who underwent HTO with and without bone grafting had a clinical and radiological union at five to six months. Spahn et al. calculated the KOOS score pre- and post-surgery [18]. They found that smokers had a KOOS score indicating poor outcomes after surgery; this was statistically significant with a p value of <0.05. But surprisingly all cases had a full union which does not completely align itself with the KOOS outcome. However, it is concluded that smoking has poor prognosis and had an odds ratio (OR) of 5.3 with 5-9 out of 5% confidence interval of 1.8-14.9. LaPrade et al. helped with proving that HTO with bone graft is a good option for the majority of smokers as four out of five ex-smokers in his study had timely union and statistically significant improved functional outcomes on IDKCS. Only one patient had non-union and was a smoker [19]. Overall we can say that bone grafting helps in union at the osteotomy site even for smokers who have poor bone healing potential.

This is further supported by the decreased need of doing any revision surgery in these patients which would indirectly lead us to believe that they had a favourable outcome. None of the patients in Passarelli et al. and Zorzi et al. had a TKR or revision of the osteotomy [16,17]. A rare exception was the one smoker in LaPrade et al. who had to undergo revision surgery due to non-union [19]. On follow-up it was noted that full union was achieved post revision. To summarise, out of the total 43 smokers in all four studies only one smoker had a failed outcome which was managed with revision surgery as stated earlier.

Discussion

The study by Passarelli et al. has good randomisation and good differentiation but significant drawbacks as explained [16]. The sealed envelope technique to aid allocation concealment is not well explained. More detail is necessary on disclosure of who sealed the envelopes and whether they were opaque or not. This could lead to bias. The best way to address this would be to use computer-generated randomisation. On the other hand it is a very good blinded trial, as all patients had iliac crest bone harvesting irrespective of whether the graft was inserted at the osteotomy site or not. This was approved by the ethics committee and patients consented for it. But it clinically seems very unethical to harvest patients' bone grafts and freeze them for the purpose of a clinical trial. It does not mention if the patients were told that their bone graft was preserved and could be used at a later date. The outcome measures used are not very valid and reliable. Also, in addition there is under-reporting of outcomes. The time for bone union has not been reported which is quite a major drawback and weakens the reliability and applicability of outcomes to the general population. Reporting of these outcomes would increase our understanding and reduce bias. The demographic variability for the sample size is significant (p<0.028). This study like other studies has a constant confounding factor in the small sample size of smokers/ex-smokers. However the overall small sample size makes external applicability poor. From the statistical analysis there was no significant difference on functional KSS scores amongst smokers who received bone grafts and those that did not (p<0.374). The patients who received bone grafts surprisingly had a worsening of knee OA as per the Ahlback radiological score (p<0.005). The cause for this worsening could not be explained in the study and neither is there any literature to support this finding. It could be attributed to the duration of OA, but there is no scientific explanation. This finding raises the issue of reporting bias and selection bias. The size of the osteotomy is <12.5 mm in all subjects. This is another confounding variable. This study does not answer if bone grafting would work in people needing bigger correction. This study could have been better reported. It would be useful to restrict use of autologous bone grafting to groups of population with a higher known risk for complications like delayed union or non-union. All patients undergoing surgery had an arthroscopy as well. The findings of arthroscopy have not been reported. This is a big limitation. Arthroscopy is a direct eyeball view to the inside of the knee. Specific details in regard to the knee articular cartilage status would be useful. This is because of the direct relation of the wear and tear of the cartilage to the symptoms of OA in any patient. An arthroscopy could detail other findings like any added pathology or impairment in other important structures like the anterior cruciate ligament (ACL) or posterior cruciate ligament (PCL). This is in relation to the extended inclusion of using HTO for ACL or PCL deficit knees. It may even be possible to explain the worsening of arthritis in some patients post-surgery if the findings on arthroscopy had been reported. Arthroscopic knee surgery can cause swelling and stiffness which can cause worsening of symptoms or exacerbation of arthritis. Also this study utilised old-generation Puddu plates for fixation which can be attributed to causing additional detrimental effects on the outcomes [23].

The study by Zorzi et al. is the most informative amongst all included studies [17]. They explain the outcomes in smokers with bone grafts and without bone grafts. This study addresses all the important outcomes like the time taken for union in smokers with and without graft and the need for conversion to knee replacement or osteotomy revision. To discuss a few drawbacks, they would be the use of first-generation plates for fixation, correction less than or equal to 12.5 mm and small duration of follow-up. The size of the osteotomy is again ≤ 12.5 mm and average size of osteotomy in the non-grafted group was 10 mm, which can act as a confounding variable impacting the results. Longer follow-up would definitely give us more relevant data and build up the evidence. Also there were confounding variables like more patients with high BMI in the grafting group, more females in the grafting group and more patients with Grade III OA changes in the group with grafting done. Though they are not statistically significant, they may have affected the outcomes in a negative way. As with other studies, the small sample size of smokers continues to be a limitation in this study as well.

Spahn et al. pointed out the prognostic factors with a good rating scale for patient selection undergoing HTO [18]. These poor prognostic factors in patients should arouse the need for bone grafting to aid healing and enhance recovery. The prognostic scoring system developed by the author is good but I feel it has probably overestimated the factors and shown that it is impossible to do HTO in these patients. Also allocation bias can be present due to unclear method of patient allocation. This can be due to poor randomisation of patients. The author has discussed the failure to follow up rate as one of the limitations of this study. KOOS was used as an outcome score in this study. Collins et al. summarised that KOOS is a very valid and reliable tool for reporting outcomes for interventions on different knee conditions thus adding value to the study as it has used good outcome measures [24]. This also minimises attrition bias. However we need to conduct a study utilising this score, calculate outcomes and then do a comparative study including those with poor prognostic criteria. Because at times the knee pain is so debilitating that doing an osteotomy for realignment and bone stock preserving osteotomy becomes a necessity.

LaPrade et al. in their case series had a 20% dropout rate [19]. The subgroup analysis of results in this study is not satisfactory. They have not reported on the outcomes of ex-smokers included in the study. The follow-up is satisfactory and the results in other subgroups are promising and have good external applicability. However they did report cases being converted to arthroplasty. This could be due to good and longer follow-up which gives more information on the longevity of HTO. The maximum follow-up was a good 8.9 years. Overall the study did not show any conflict of interest but had a low level of evidence (IV). To mitigate as many confounding factors as possible, the need is to conduct a randomised trial with longer follow-ups and double blinding of patients and investigators, with standardised surgical implants and techniques. There should be an inclusion of patients needing greater corrections.

The completed quality assessments for Jadad (Table 4), CASP (Table 5), STROBE (Table 6) and Delphi (Table 7) of the included studies can be found in the Appendices.

The limitations of this study are that we have used narrative synthesis owing to the small sample size of target population i.e. smokers or ex-smokers and the heterogeneity in study design i.e. a mix of RCT, case series and a non-randomised study. Thus, there are no statistical figures or numbers which would quantify the impact of results. This disabled the use of any statistical tool like forest plot or I square to be used in this review. All studies have used Puddu plates for fixation of osteotomy site. The choice of a better alternative in plates would be ideal. None of the studies include size of the osteotomy of more than 12.5mm. This limits the application of this review to patients who need a greater correction.

Further scope could be a pilot study performed in a small number to see if it really works followed by performing an RCT would be the best way to find out the results of bone grafting in the general population including smokers. This would be high-level evidence. However there will always be limitations due to blinding issues, confounding variables like age, sex, BMI, previous status of OA and ligamentous injury along with previous surgery. These variables could be minimised by utilising stringent inclusion criteria and a good pre-operative evaluation of patients. The use of Tomofix plates, the new generation locking plates, help in reducing the need for graft and also strengthen the construct for osteotomy below 12.5 mm size thereby improving outcomes. Conducting a meta-analysis would carry high level of evidence for this topic of interest - studies that give results of HTO in smokers without bone grafting for ≥12.5 mm osteotomy size.

## Conclusions

HTO with bone grafting in smokers seems to be promising with the osteotomy size ≤12.5 mm with first-generation plates. However there is lack of evidence for its application in osteotomies more than 12.5 mm using the recent advanced locking plating system. The preferred choice of bone graft would be cancellous iliac crest auto - grafts bearing in mind the graft site morbidity. Other choices would include allografts but these are subject to a risk of viral infection transmission like hepatitis C, human immunodeficiency virus (HIV-1), and human T-cell leukaemia virus and are not cost-effective. There is need for more high evidence-based studies to address this uncertainty and improve our understanding. Ideally, cessation of smoking would be the best measure against poor outcomes after surgery and would mean encouraging healthy lifestyle measures. Smoking cessation is not easy but offering mental health support would aid in preventing smoking relapse. 

## Appendices

Quality assessments of studies

**Table 4:** Jadad scoring of randomised studies

Jadad score	Study	Study
Checklist	Passarelli et al. [16]	Zorzi et al. [17]
Was the study described as randomised?	Yes	Yes
Was the method used to generate randomisation described and appropriate?	Yes	Yes
Was the study double blinded?	Yes	Yes
Was the method of blinding described and appropriate?	Yes	Yes
Was there a description of withdrawals and drop-outs?	No	No
Results	4/5 High range of quality score	4/5 High range of quality score

**Table 5:** Checklist for quality assessment of randomized studies included in the study (Critical Appraisal Skills Program [CASP])

No	Sections	Passarelli et al. [16]	Zorzi et al. [17]	Explanations
1	Clear focussed question addressed	Yes	Yes	Title includes a much focussed clinical question.
2	Randomization	Yes	Yes	Well explained.
3	Intention to treat principle followed	No	No	Drop outs were excluded.
4	Blinding	Double	Double	Patients and investigators were blinded.
5	Demographic similarity of patients	Yes	Yes	Almost equal number of patients and subgroups.
6	Equal treatment apart from intervention	Yes	Yes	All patients received same post-operative treatment.
7	Specific Outcomes	Yes	Yes	Knee scores, bone union, radiological evaluation etc.
8	Precision of reporting	Partially explained	Yes	P values tabulated, Confidence limits.
9	External validity	Good	Good	Patient demographics but apparent small sample size in spite of power calculation.
10	All outcomes reported	Yes	Yes	
11	Harm and risk benefits	Beneficial	Beneficial	Increasing smoking young population with early knee OA is concerning and benefits outweighing the risks.

**Table 6:** Strengthening the Reporting of Observational studies in Epidemiology (STROBE) checklist for quality assessment of Spahn et al. Adapted from Spahn et al. [18] HTO: high tibial osteotomy, KOOS: Knee injury and Osteoarthritis Outcome Score

Domain	Observations	Location in article
Title and abstract	Title does not explain the type of study but abstract is well presented and cover all important outcomes and conclusion of the study. Introduction of this study being of retrospective type is done.	Page 1 in abstract and the methods section. Type of study mentioned in the introduction Page 1.
Introduction/Background	Well explains the need for study and gives scientific background.	Page 1 introduction paragraph.
Objectives	To study if significant factors affecting outcome of HTO could be delineated.	Page 1 in introduction.
Study design	Introduction of this study being of retrospective type is done.	Type of study mentioned in the introduction Page 1.
Ethical consideration	Ethical issues have not been addressed at all.	N/A
Outcomes	KOOS scores in smokers did not rise post operatively when compared with non-smokers. Smoking was a poor prognosticating factor for outcomes with Odds Ratio 4.3 and CI 1.8-14.9	Table 2 given on page 193.
Bias	No consideration of study limitations been done or presented.	N/A
Population	Specific inclusion and exclusion criteria set out.	Page 190-191 under methods heading.
Key results	Prognostic scoring system could be developed for outcomes following HTO.	Page 191 and 193.
Generalizability	The sample size is adequate and has good external validity.	Page 193-195.
Conflict of interest	No mention has been made.	N/A.
Strengths	Not mentioned or highlighted.	N/A.
Limitations	Not mentioned or highlighted.	N/A.
Drop outs/Failed follow up	Have been clearly stated with reasons.	Page 191 under follow up and complications.
Complications	Clearly stated.	Page 191.

**Table 7:** Quality Appraisal Delphi checklist for LaPrade et al. Adapted from LaPrade et al. [19] OA: Osteoarthritis, MOWTO: Medial Open Wedge Tibial Osteotomy

No	Domain		Explanation & location in article
1.	Was the hypothesis/aim/objective of the study clearly stated?	Yes	Young and middle-aged people with medial compartment of knee OA have significant improvement in scores after MOWTO. Page 2 before methods.
2.	Was the study conducted prospectively?	Yes	All patients from May 2000-July 2007 were followed prospectively. Page 2 under methods.
3.	Were the cases collected in more than one centre?	Unclear	Unclear.
4.	Were patients recruited consecutively?	Yes	All patients from May 2000-July 2007 were followed prospectively. Page 2 under methods.
5.	Were the characteristics of the patients included in the study described?	Yes	Patient’s demographics clearly tabulated. Page 6 under results.
6.	Were the eligibility criteria (i.e. inclusion and exclusion criteria) for entry into the study clearly stated?	Yes	Strict inclusion and exclusion criteria, though they excluded active smokers, they have included ex-smokers. Page 2 under methods.
7.	Did patients enter the study at a similar point in the disease?	Yes	All patients which fitted in the inclusion criteria were studied and followed up.
8.	Was the intervention of interest clearly described?	Yes	Surgical technique clearly described on page 3 under operative technique.
9.	Were additional interventions (co-interventions) clearly described?	Yes	Patients had arthroscopy before surgery in few cases. Page 3 under operative technique.
10.	Were relevant outcome measures established a priority?	Yes	Specific outcome measures have been selected and results displayed in detail. Page 6 under results.
11.	Were outcome assessors blinded to the intervention that patients received?	Unclear	No clear mention has been made on who followed the patients post-operatively and who were the investigators.
13.	Were the relevant outcome measures made before and after the intervention?	Yes	Clear mention of scores has been made pre and post operation. Page 6 under results heading.
14.	Were the statistical tests used to assess the relevant outcomes appropriate?	Yes	Page 6 under heading statistical analysis.
15.	Was follow-up long enough for important events and outcomes to occur?	No	Short follow up of average 2 years was one of the limitations of this case series. Page 10 just prior to conclusion paragraph.
16.	Were losses to follow-up reported?	Yes	Clearly reported in detail on page 6 under demographics sub-heading.
17.	Did the study provided estimates of random variability in the data analysis of relevant outcomes?	Partial	Page 6 under heading of results.
18.	Were the adverse events reported?	Yes	Explained well under heading of complications page 7.
19.	Were the conclusions of the study supported by results?	Yes	Page 10 under conclusion. Clearly states that outcomes are improved after mean 3.6 years follow up. Page 10 under conclusion.
20.	Were both competing interests and sources of support for the study reported?	Yes	Stated clearly on page 1.
